# Cannabichromene Induces Neuronal Differentiation in NSC-34 Cells: Insights from Transcriptomic Analysis

**DOI:** 10.3390/life13030742

**Published:** 2023-03-09

**Authors:** Andrea Valeri, Luigi Chiricosta, Simone D’Angiolini, Federica Pollastro, Stefano Salamone, Emanuela Mazzon

**Affiliations:** 1 IRCCS Centro Neurolesi “Bonino-Pulejo”, Via Provinciale Palermo, Contrada Casazza, 98124 Messina, Italy; 2 Department of Pharmaceutical Sciences, University of Eastern Piedmont, Largo Donegani 2, 28100 Novara, Italy; 3 Plantachem S.r.l.s., Via Amico Canobio 4/6, 28100 Novara, Italy

**Keywords:** cannabichromene, neuronal differentiation, NSC-34, transcriptomic analysis

## Abstract

Phytocannabinoids, with their variety of beneficial effects, represent a valid group of substances that could be employed as neurogenesis-enhancers or neuronal differentiation inducers. We focused our attention on the neuronal-related potential of cannabichromene (CBC) when administered to undifferentiated NSC-34 for 24 h. Transcriptomic analysis showed an upregulation of several neuronal markers, such as *Neurod1* and *Tubb3*, as well as indicators of neuronal differentiation process progression, such as *Pax6*. An in-depth investigation of the processes involved in neuronal differentiation indicates positive cytoskeleton remodeling by upregulation of *Cfl2* and *Tubg1*, and active differentiation-targeted transcriptional program, suggested by *Phox2b* and *Hes1.* After 48 h of treatment, the markers previously examined in the transcriptomic analysis are still overexpressed, like *Ache* and *Hes1*, indicating that the differentiation process is still in progress. The lack of GFAP protein suggests that no astroglial differentiation is taking place, and it is reasonable to indicate the neuronal one as the ongoing one. These results indicate CBC as a potential neuronal differentiation inducer for NSC-34 cells.

## 1. Introduction

*Cannabis sativa* L. is a plant belonging to the *Cannabaceae* family that has been widely used, since ancient times, for both recreational and medical purposes [1]. *C. sativa* contains different terpeno-phenolic compounds called cannabinoids [2,3]. 

Recently, cannabinoids added to their pool of properties also neuronal differentiation induction and neurogenesis promotion [4,5]. The discovery of brain areas, also in humans, where stem cells are present and can efficiently differentiate opened a new era of possible therapeutic strategies. What was thought to be irreplaceable became able to self-renew [6], and different studies were made to investigate how neurogenesis discovery could be exploited [7]. The association between cannabinoids and neurogenesis or cannabinoids and neuronal differentiation is quite recent but very promising. 

Although the attention is focused primarily on the psychoactive delta-9-tetrahydrocannabinol (Δ^9^-THC) and the non-psychoactive cannabidiol (CBD), the scientific community is recently considering the potentially therapeutic properties of lesser abundant phytocannabinoids such as cannabichromene (CBC) [8]. Cannabichromene is a non-psychoactive cannabinoid derived by the decarboxylation of cannabichromenic acid (CBCA), induced by the enzymatic CBCA synthase action [9]. 

The plethora of biological effects induced by the properties of phytocannabinoids, and also by the effects that CBC exerts on adult neural stem progenitor cells (NSPCs), leading them to sustain their viability and differentiation [10], brought to our attention the possibility to use this compound on NSC-34 cell line. It is a hybridoma between spinal cord cells, taken from embryos of mice, and neuroblastoma. They undergo differentiation when exposed to retinoic acid and resemble the features of motor neurons as follows: they generate action potentials, and they synthetize acetylcholine, as well as store it and release it [11,12]. Indeed, NSC-34 proved to be a good model to investigate neuronal differentiation and development. To investigate the potentialities of our cannabinoid on neurons, we treated undifferentiated NSC-34 for 24 h and 48 h with 10 µM of CBC. The transcriptomic profile was inspected, as well as protein levels with Western blot analysis, to evaluate if CBC can induce neurogenesis or can trigger the differentiation process.

## 2. Materials and Methods

### 2.1. Extraction, Isolation, and Purification of CBC from C. sativa

The cannabichromene was provided by Plantachem S.r.l.s. (Novara, Italy) with a purity of 94%.

**Figure 1:**
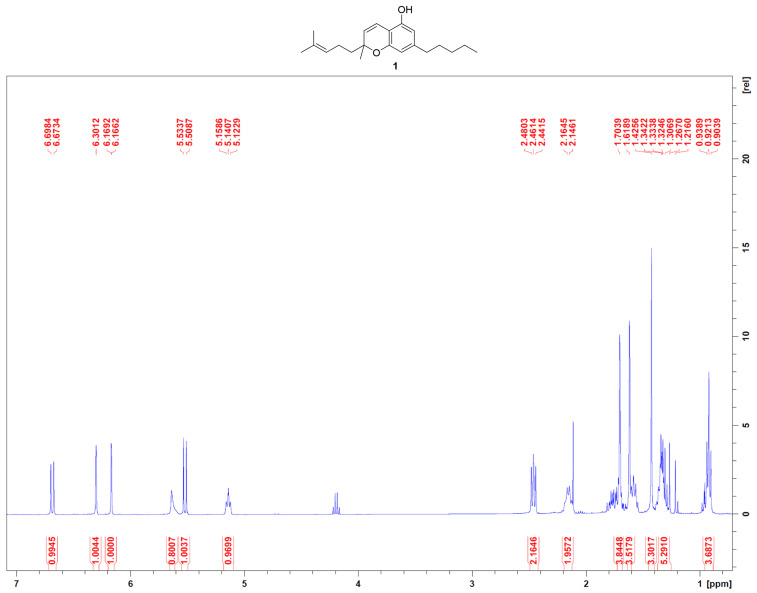
^1^H NMR of CBC (**1**) in CDCl_3_, 400 MHz.

Nonwoody aerial parts, inflorescences, and leaves were extracted with acetone affording a dark green syrup that was heated at 130 °C under stirring for 45 °C in a paraffine bath to achieve the decarboxylation of cannabinoid acids. The reaction was followed by TLC (silica, petroleum ether-EtOAc 80:20). This latter decarboxylated fraction was fractionated by low-pressure chromatography (LPC) on silica gel (250 g, petroleum ether-EtOAc gradient from 90:10 to 20:80) to afford three fractions (I, II, and III). Fraction I (1.345 g) was further purified with low-pressure chromatography (LPC) on silica gel (75 g, petroleum ether-EtOAc gradient from 90:10 to 80:20) to afford 96 mg of cannabichromene as brownish powder, which structure has been identified according to ^1^H NMR reported in the literature [13]. The chemical formula of CBC and ^1^H NMR are reported in Figure 1.

^1^H 400 MHz NM spectra were measured on Bruker 400 spectrometers (Bruker^®^, Billerica, MA, USA). Chemical shifts were referenced to the residual solvent signal (CDCl_3_: δ_H_ = 7.26). Silica gel 60 (70–230 mesh) used for low-pressure chromatography was purchased from Macherey-Nagel (Düren, Germany). Purifications were monitored by TLC on Merck 60 F254 (0.25 mm) plates, visualized by staining with 5% H_2_SO_4_ in EtOH and heating. Chemical reagents and solvents were from Aldrich (Darmstadt, Germany) and were used without any further purification unless stated otherwise.

### 2.2. Cells Culture and Treatment

NSC-34 was provided by Cedarlane Corporation (Burlington, ON, Canada). The maintenance medium was composed as follows: DMEM High Glucose, 10% Fetal Bovine Serum, 1% penicillin/streptomycin, and 1% L-Glutamine. All reagents were bought from Sigma-Aldrich, Merck KGaA (Darmstadt, Germany).

When the cells reached about 80% confluence, they were seeded in 6-well plates for obtaining material for transcriptomic analysis, in 96-well plates for MTT test and in 25 cm^2^ flasks for further Western blot analysis. The seeding concentration was 200,000 cells/mL. The first group was used as control (CTRL), while the second one underwent medium change with fresh medium +10 µM of CBC (CBC). CBC was dissolved in DMSO, which final concentration was inferior to 0.1%. CTRL underwent a complete medium change with medium without CBC, in order to maintain the same conditions in terms of nutrients and cell stress. In total, 24 h after the treatment, the morphology and conditions of the cells were checked and pictures from each group were taken using light microscopy (LEICA DM 2000 combined with LEICA ICC50 HD camera, Wetzlar, Germany). Then, all wells from the 6-well plates were harvested for further analyses. Cells in 96-well plates underwent MTT test. The same procedure was performed for an additional set of 6-well plates to obtain the material for transcriptomic analysis and 25 cm^2^ flasks to obtain material for Western blots. The mediums were changed after 24 h. Cells were harvested after 48 h to undergo transcriptomic and Western blot analysis. Experiments were carried out at three independent and different times.

### 2.3. MTT Test

In total, 24 h after the treatment with 10 µM of CBC, the medium was replaced in all wells with fresh medium with MTT at a concentration of 0.5 mg/mL (Sigma-Aldrich Merck KGaA (Darmstadt, Germany). After 4 h at 37 °C, the presence of crystals was visible at the bottom of each well. The crystals were resuspended using acidic propanol and the optic density was measured with a spectrophotometer (570 nm).

### 2.4. Total RNA Extraction, Library Preparation and Transcriptomic Analysis

Cells from the 6-well plates were harvested for RNA extraction. RNA extraction from cell pellets proceeded as manufacturer’s instruction from the Maxwell^®^ RSC simplyRNA Cells Kit (Promega, Madison, WI, USA). Library was prepared using TruSeq RNA Exome protocol (Illumina, San Diego, CA, USA) [14] and analyzed by Illumina instrument Miseq.

### 2.5. Bioinformatic Analysis

To check the quality of the reads used for the analysis presented in this work, we used fastqc v0.11.9 (Babraham Institute, Cambridge, UK) [15]. After the quality check, we removed the adapters and the reads with a bad quality score using Trimmomatic v. 0.40-rc1 (Usadel Lab, Aachen, Germany) [16]. The reads that passed the filter were aligned to a reference genome using Spliced Transcripts Alignment to a Reference (STAR) RNA-seq aligner 2.7.10a_alpha_220207 (New York, NY, USA) [17]. The genome used to align the reads is the primary annotation of mouse genome v. M28 from gencode. After the alignment, phase we obtained the count of transcripts for the genes using htseq-count v. 0.13.5 [18]. Using count of the transcripts was possible to perform analysis of differentially expressed genes using DESeq2 library v. 1.36.0 [19] through the programming language R v. 4.2.0 (R Core Team, Vienna, Austria). Benjamini-Hochberg correction was used to reduce the number of false positive DEGs setting the threshold of q value to 0.05. With the list of the DEGs we obtained the enriched pathways and ontologies using the package for R, clusterProfiler v. 4.4.3 [20]. Through the inspection AmiGO2 [21], we studied the enriched ontologies.

### 2.6. Western Blot Analysis

Cells from the 25 cm^2^ flasks were harvested using trypsin-Ethylenediaminetetraacetic acid (EDTA). The resulting pellet underwent protein extraction using NE-PER™ Kit for Nuclear and Cytoplasmic Extraction Reagents (Thermo Scientific™, Waltham, MA, USA) following manufacturer protocol, as previously described [22]. In total, 25 µg of proteins were separated using sodium dodecyl sulfate-polyacrylamide gel electrophoresis (SDS-PAGE) after quantification by Bio-Rad Protein Assay (Bio-Rad Laboratories, Hercules, CA, USA) using bovine serum albumin (BSA) as the standard. Proteins were then transferred to a PVDF transfer membrane (Immobilon-P PVDF, Merck Millipore division of Merck KGaA, Darmstadt, Germany). The membranes were incubated at room temperature using skimmed milk (5% in TBS). After 1 h, membranes were incubated overnight at 4 °C with the following primary antibodies:

- GFAP (GA5): 1:1000 (#3670), Cell Signaling Technology, Danvers, MA, USA;
- AChRα1 (153): 1:500 (sc-65829) Santa Cruz Biotechnology, Inc., Dallas, TX, USA;
- GAPDH (14C10): 1:1000 (#3683), Cell Signaling Technology, Danvers, MA, USA.

The membranes were then incubated 1 h at room temperature with the according secondary antibody as follows:

- Chicken anti-rat IgG-HRP: 1:2000 (sc-2956) Santa Cruz Biotechnology, Inc., Dallas, TX, USA;
- Chicken anti-mouse IgG-HRP: 1:2000 (SA1-72021) ThermoFisher Scientific, Rockford, IL, USA.

The different bands were visualized using Luminata Western HRP Substrates, Millipore Corporation, Billerica, MA, USA), and obtained and quantified with ChemiDoc™ MP System (Bio-Rad Laboratories S.r.l., Hercules, CA, USA).

### 2.7. Statistical Data Analysis

MTT test results and ImageJ quantification of Western blot bands underwent statistical analysis using GraphPad Prism version 6.0 software (GraphPad Software, La Jolla, CA, USA). The data were statistically analyzed by Student’s *t*-test to compare the two groups and one-way ANOVA when the groups were more than two. When *p*-value was equal to or less than 0.05, it was considered statistically significant. 

## 3. Results

### 3.1. MTT Test and Evaluation of Cells Morphology

The possible toxicity of CBC was checked. NSC-34 cells were incubated for 24 h with 10 µM of CBC before undergoing the MTT test. Previous experiments already tested the DMSO group, and, as a result, a concentration below 0.1% is already known for not being toxic to cells [23].

**Figure 2:**
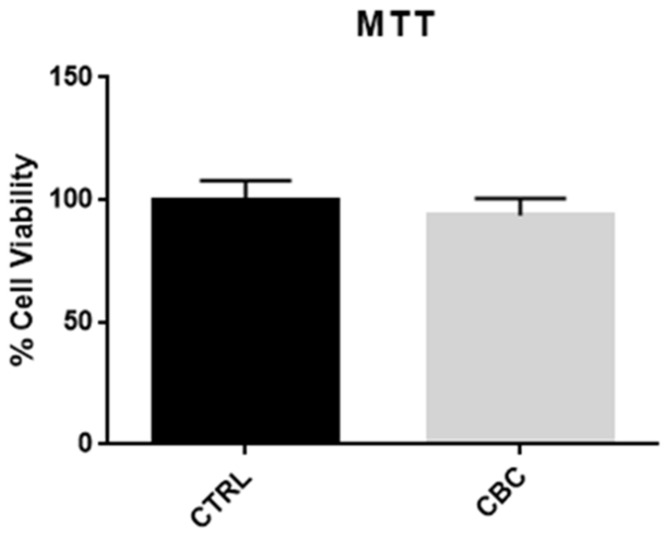
MTT test after 24 h of CBC treatment. There is no significant difference between the control group and the treated group, thus proving that CBC at 10 µM is not toxic for the cells.

As shown in Figure 2, the difference in cell viability is not significant.

**Figure 3:**
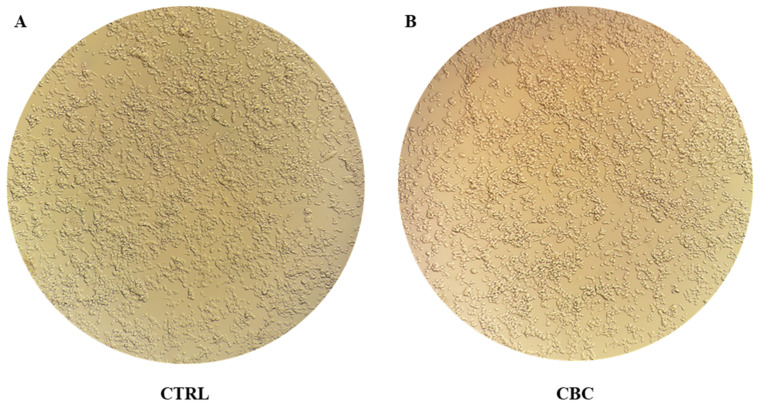
Pictures of the two groups 24 h after (**A**) completing fresh medium change (CTRL) and (**B**) 10 µM CBC treatment (CBC). No difference in cell density indicates that cells are not under stress or they did not undergo apoptosis during treatment, as suggested by the MTT test.

Cell morphology was inspected to evaluate their status. As can be seen in Figure 3, there is no visual difference in cell morphology between the two groups and, as supported by MTT data, it can be concluded that CBC is not harmful to the cells.

### 3.2. Inspection of the Transcriptomic Profile

The transcriptomic analysis performed on CBC24h in comparison to CTRL revealed 4985 differentially expressed genes (DEGs), among which 2567 DEGs were upregulated while 2418 were downregulated in CBC24h. On the other hand, CBC48h had 2814 upregulated and 3038 downregulated DEGs (for a total of 5852 DEGs).

We enriched the whole set of DEGs for biological process terms of Gene Ontology for both times of CBC. We particularly observed, “stem cell differentiation” (GO:0048863), “neural crest differentiation” (GO:0014033), “cell differentiation in hindbrain” (GO:0021533), “negative regulation of necrotic cell death” (GO:0060547), “negative regulation of cell cycle process” (GO:0010948), “negative regulation of cell cycle phase transition” (GO:1901988), “negative regulation of mitotic cell cycle phase transition” (GO:1901991) terms related to neuronal differentiation behavior and enriched both for CBC24h and CBC48h. The seven aforementioned biological process terms are highlighted in the bubble plot in Figure 3. The highest scores are obtained in CBC48h for GO:1901991, GO:1901988 and GO:0010948. Conversely, GO:0060547 have a higher score in CBC24. Curiously, GO:0021533 and GO:0014033 had almost identical scores.

**Figure 4:**
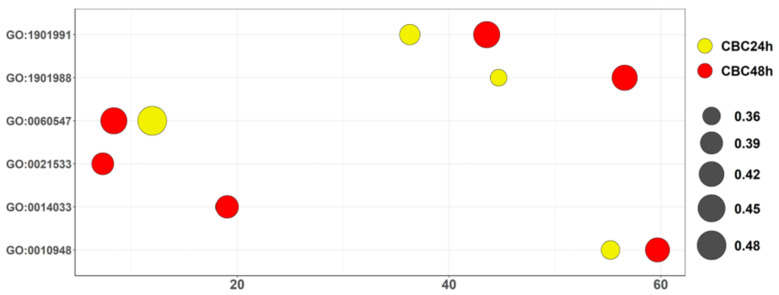
Bubble plot of biological process terms of Gene Ontology. All the terms represented in the vertical axis by ID are enriched in our analysis bot for CBC 24 h and CBC 48 h. The score in the horizontal axis is obtained as −log_2_ (q-value). The size of the bubbles is the ratio computed as the number of DEGs included in the ontology over the total genes of the ontology itself. Ontology highlighted in yellow was found in CBC 24 h while in red in CBC 48 h.

**Figure 5:**
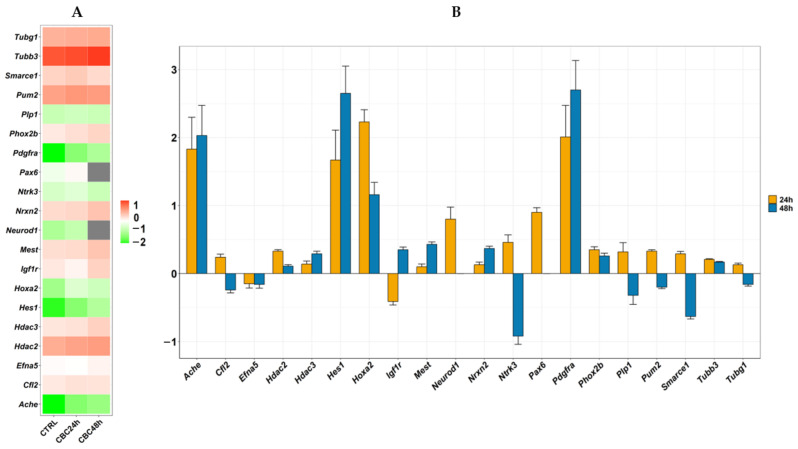
Expression of markers of neurodifferentiation. Frame (**A**) shows a heatmap related to level of expression of the markers in CTRL or CBC at 24 h or 48 h. The mean level of expression among the markers is in white so that DEGs expressed more than the mean go towards the red palette while DEGs expressed lower than the mean is represented in the green palette. In grey the genes that are no DEG in the comparison against CBC 48 h. Frame (**B**) shows the fold change of markers so that DEGs with positive values are upregulated while DEGs with negative values are downregulated in CBC. For each bar is included the standard error bar.

Neurons, like other cells, express typical markers during their differentiation process and during the stage through which they have to pass before becoming mature cells. The relevant markers for our study are shown in Table 1 and Figure 4 also include the expression levels or the fold change of markers identified. In order to evaluate the markers with higher relevance in the study, we plotted in Figure 5A a heatmap, in which are represented their expression level. In detail, we corrected the value through a z-score normalization after a log_2_ transformation to clearly visualize the markers with expression levels higher (in red) or lower (in green) than the mean value. *Hdac2*, *Tubg1*, *Pum2*, *Tubb3*, *Hdac3*, *Cfl2*, *Phox2b*, *Nrxn2*, *Mest* and *Smarce1* have a level of expression clearly higher than the mean in both groups, while *Ntrk3*, *Plp1*, *Hoxa2*, *Neurod1*, *Hes1*, *Pdgfra1* and *Ache* have the same behavior with values lower than mean. *Igf1r*, *Efna5* and *Pax6* have expressions slightly over the mean value. Additionally, in Figure 5B, we reported the fold change of the same markers obtained for CBC in comparison with CTRL. It is clear that most DEGs result in upregulation in the CBC group. 

### 3.3. Western Blot Analysis

**Figure 6:**
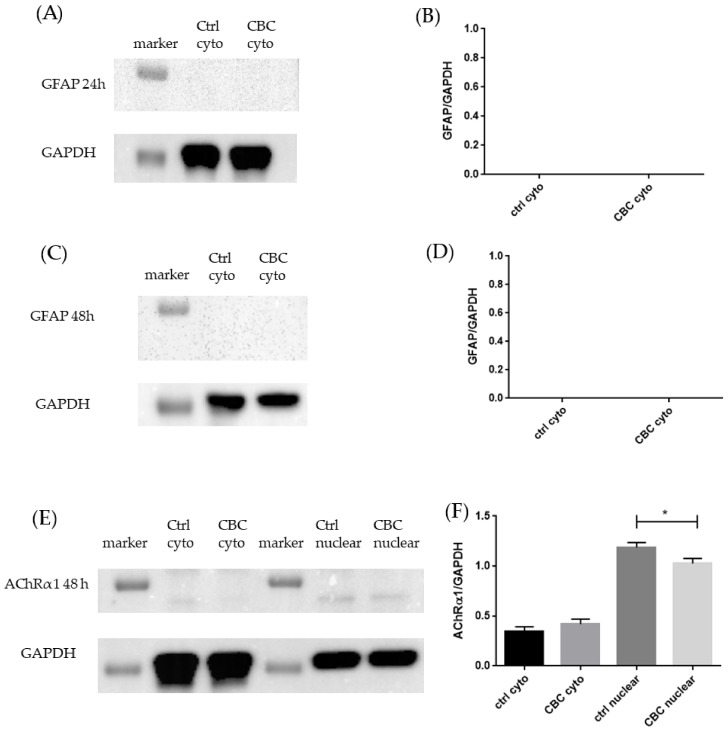
(**A**) Evidence of the absence of GFAP after 24 h of CBC exposure and GAPDH used to confirm the protein’s presence; (**B**) Densitometric analysis of GFAP after 24 h of CBC exposure; (**C**) Evidence of the absence of GFAP after 48 h of CBC exposure and GAPDH used to confirm the protein’s presence; (**D**) Densitometric analysis of GFAP after 48 h of CBC exposure; (**E**) Evidence of the presence of AChRα1 after 48 h of CBC exposure and GAPDH used to confirm the protein’s presence; (**F**) Densitometric analysis of AChRα1 after 48 h of CBC exposure, * *p* < 0.05.

The evaluation of the possible differentiation of NSC-34 cells after CBC exposure was tested using different markers. GFAP evaluation was used to exclude a possible differentiation in glia and astrocytes, and, as seen in Figure 6A–D, NSC-34 are not proceeding in such a way.

The receptor of acetylcholine, AChRα1, was not present neither in the cytoplasm nor in the nucleus after 24 h, while it is present after 48 h both in the control group and in CBC treated group, as shown in Figure 6E,F.

## 4. Discussion

Phytocannabinoids and their influence on neuronal fate are fascinating fields of study. This capacity makes them suitable candidates to explore the neurogenesis and the neuronal differentiation process in an in vitro model such as NSC-34. 

After exposing undifferentiated NSC-34 to 10 µM of CBC, its toxicity and influence on neurogenesis or neuronal differentiation were investigated. The results from the MTT test, as shown in Figure 2, suggest that treatment with 10 µM of CBC does not decrease the cell viability after 24 h. The lack of significance in the comparison between control and treated cells indicates that the tested phytocompound is not toxic at the dose and time of treatment. Figure 3 gives an overview of the cell’s status after treatment, and since there is no clear difference in morphology between the two groups, it can be concluded that the phytocompound does not represent a source of stress for the cells.

Given these statements, the aim of our study was to investigate whether CBC can initiate this process. The graphs reported in Figure 5 gave us an indication of the main genes that will be inspected and further discussed.

*Igf1r* encodes for Insulin-like growth factor 1 receptor, and, in our data, its expression is reduced after CBC treatment. The main ligand of this receptor is insulin-like growth factor 1 (IGF-1), and it can be produced by the liver, but also by neurons in a growth hormone-independent way [24]. IGF-1 and its receptor trigger the signals of the phosphoinositide 3-kinase (PI3K) pathway, which is known to promote cell survival but also proliferation [25]. The decreased expression of *Igf1r* in NSC-34 as a consequence of CBC treatment should be interpreted as a reduction in proliferation. *Pdgfra* encodes for platelet-derived growth factor receptor alpha, and CBC strongly upregulates it. *Pdgfra* is one type of receptor for platelet-derived growth factors (PDGF), and, in particular, PDGFA and PDGFB heterodimer is necessary for the growth and maturation of stem cells. PDGFA receptor is expressed in embryonic ectomesenchymal-derived cells, and common progenitors from neural crest seem to differentiate in both neural and mesenchymal cells. In regards to adult neural progenitors, PDGFRA, along with PDGFRB, appear to play a role in cell survival [26]. Its upregulation is in favor of cell survival, which correlates very well with MTT data. *Ntrk3* encodes for neurotrophic receptor tyrosine kinase 3 (TrkC), and it is upregulated in NSC-34 after CBC exposure. It is the receptor of neurotrophin 3 (NT-3), and together they play an important role in the development of the nervous system. Indeed, NT-3 can increase the expression of its receptor, and this can trigger the differentiation in NSCs. In transfected cells transplanted in a model of spinal cord injury, NT-3 and TrkC were able to cooperate in order to restore the lost neuronal connectivity [27]. Considering that NSC-34 origin is in the spinal cord of mice, the increase in this receptor in NSC-34 after CBC treatment suggests that the differentiation of NSC-34 might be efficiently triggered.

*Cfl2* encodes for cofilin-2, and it is overexpressed in NSC-34 after CBC treatment. *Cfl2* is part of a protein family that interacts with a major component of the cytoskeleton, actin. The expression of *Cfl2* in neuronal progenitor cells is usually very low [28], but it has been found that conditional knockout of *Cfl2* influences axonal branching and growth during regeneration [29]. It is coherent to think that it may have an important role in cytoskeleton remodeling, occurring during axonal growth. Its overexpression indicates that this remodeling is taking place, and it is reasonable to think that the aim is neuronal differentiation. Other markers of cytoskeleton remodeling with neuronal differentiation as an aim support this statement. *Tubg1* encodes for tubulin gamma 1, and it is overexpressed in NSC-34 after CBC exposure. Gamma tubulins are a superfamily of proteins involved in the microtubule’s nucleation. *Tubg1* is expressed ubiquitously in the tissues of the body. Even though isotype 2 is specific to the brain, *Tubg1* appeared to be more expressed than isotype 2 in the mature brain [26]. *Tubb3* encodes for Tubulin β 3 Class III, and it is upregulated by CBC in NSC-34. It is a microtubule protein, neuron-specific, and it is commonly used as a marker of differentiated neurons. *Tubb3* is expressed by NSC-34 both in undifferentiated and differentiated forms [30]. Its expression is distributed in the developing neural plate, so its role is not limited to being a marker, but it also actively regulates the neuron-related developing process [31]. The increased expression of *Tubg1* and *Tubb3* after CBC treatment points toward the cytoskeleton remodeling directed to neuronal differentiation.

*Mest* encodes for mesoderm-specific transcript, and it is slightly upregulated in NSC-34 following CBC treatment. Its expression is correlated with dopaminergic neuron maturation. As demonstrated by knock-out mice, *Mest* is also involved in the survival of adult dopaminergic neurons in substantia nigra [32], while the hypermethilation of its promoter was found in Alzheimer’s Disease patients’ brains. In vitro, the depletion of *Mest* resulted in the block of neuronal differentiation [33]. These results indicate the important role played by *Mest* in the neuronal differentiation process; thus, its increment after the CBC treatment suggests a correct progress toward the differentiated status.

*Hdac3* is overexpressed is in NSC-34 after CBC exposure. *Hdac3* encodes for the protein histone deacetylase-3, which is a member of the histone deacetylases family, and, as other members like -1 and -2, they are mainly expressed in the nucleus. The action of HDACs is the opposite of the histone acetyltransferases (HTAs) family since the first one represses gene transcription while the second allows the relaxation of chromatin and enhancement of transcription [34]. Evidence from developing mice stated that *Hdac3* is essential for the correct formation of the brain since *Hdac3* deletion results in fetal lethality. In humans, mutations in *HDAC3* lead to intellectual disability. Indeed *Hdac3* is highly expressed during the development of the cerebral cortex in mice, and it is also required for neuronal progenitor cells [35]. *Hdac2* is upregulated as well after CBC treatment in NSC-34 cells. As *Hdac3*, it is mainly expressed and localized in the nucleus of the cells, where it exerts its role as a transcriptional regulator. The conditional deletion of *Hdac2* resulted in defects in neuronal development, indicating its essential role in this process, as follows: *Hdac2* knockdown blocks neuronal maturation and leads the progenitor to death. Interestingly, this does not happen to astrocytes, indicating how *Hdac2* might regulate the differentiation in a more refined way [36]. The overexpression of both *Hdac3* and *Hdac2* suggests that the differentiation is probably proceeding without defects. *Hoxa2* encodes for Homeobox A2, and it is upregulated in NSC-34 after CBC treatment. Its action is correlated with *Pax6*, encoding for Paired Box 6, whose expression is upregulated as well after CBC treatment. *Hox* and *Pax* genes are transcription factors that are essential during early neurogenesis, but they are also important during differentiation [37]. *Pax6* plays its role in CNS development, acting upstream of genes networks involved in the formation of neural circuits and neuronal migration. What is very interesting about *Pax6* expression is that the activation of genes involved in neuronal development happens together with the repression of genes involved in mesodermal and endodermal development [38]. For its specificity toward neuronal maturation, its upregulation after CBC treatment points to a neuronal differentiation fate for NSC-34. 

*Hoxa2*, along with *Hoxb2*, regulates the expression of several genes involved in the specification of neuronal subsets, and one of these is *Phox2b*. *Phox2b* encodes for paired-like homeobox 2B, and it is upregulated in our experimental set. *Phox2b* is a transcription factor and, along with *Phox2a*, is expressed only in a certain population of neuronal progenitors. Even though they are expressed both in the central and peripheral nervous system, what is interesting is that they are restricted to motor neurons, both branchiomotor and visceral motor ones. When *Phox2b* is not correctly expressed, neuronal differentiation is not progressing as it should. *Phox2b* is able to control the differentiation at multiple levels, both general and sub-type specific. This means that *Phox2b* helps in the general neuronal differentiation of the progenitors, but it is also necessary to guide them toward branchiomotor and visceral motor neurons fate [39]. Its upregulation after CBC treatment to NSC-34 is an indication of correct differentiation in motor neurons, as is expected from NSC-34 cells. *Hes1* encodes for Hes Family BHLH Transcription Factor 1, and it is strongly upregulated after CBC treatment in NSC-34 cells. *Hes1* is a transcriptional repressor, so its role is to block the expression of genes, but its knockdown appears to induce premature differentiation of neurons when their number is not enough to sustain mature brain activity. On the other hand, the overexpression of *Hes1* represents an obstacle to proliferation. The explanation lies in the oscillatory expression of *Hes1*, which correctly balances proliferation and differentiation [40]. In the case of NSC-34 treated with CBC, *Hes1* overexpression is coherent with the proliferation stop.

*Nrxn2* encodes for neurexin-2, and it shows upregulation in our experiment after CBC treatment. *Nrxn2* is important at the pre-synaptic membrane levels since it is involved in the release of neurotransmitters, and it is one target in the clinical development of spinal muscular atrophy. It was clear that *Nrxn2* loss resulted in a reduction in axon excitability in motor neuron cells [41]. This evidence suggests that *Nrxn2* has a very important role involving the correct functioning of motor neurons. Its upregulation after CBC treatment could indicate the preparation of NSC-34 to exert their role as motor neuron-like cells. *Pum2* encodes for Pumilio2, and it was found upregulated after CBC treatment. *Pum2* is a protein with important functions as RNA binding protein, thus regulating a variety of cellular processes. One of these is neuronal differentiation. Indeed, *Pum2* is involved in axon branching, dendritic processing formation, and, as a consequence, synapse maturation. In detail, it seems that *Pum2* is able to regulate soma size, the so-called “body” of neurons from which axons and dendrites grow. This was clear in an experiment where *Pum2* was selectively downregulated, and it resulted in a reduced soma size. Moreover, *Pum2* also plays an important role in regulating the excitability and inhibition of neuronal cells, indicating its influence on the maturation of neurons [42]. Its upregulation after CBC exposure suggests that NSC-34 are pushed on the way to becoming differentiated neurons. *Smarce1* encodes for SWI/SNF-related, matrix-associated, actin-dependent regulator of chromatin, subfamily E, member 1 and it is upregulated in our study. *Smarce1* protein, BAF-57, is part of a much larger complex called SWI/SNF complex, whose functions include the facilitation of genes expression [43]. Notably, BAF57 is highly present in hippocampal neurons, while in glia, it is low, indicating its specificity for neuron cells. Its knock down resulted in dendritic growth defect, suggesting its role in the regulation of their shape [44]. The upregulation of *Smarce1* in our experimental set suggests the capacity of CBC to influence the regulation of dendritic shape and, along with the other genes, appear to be able to regulate a different aspect of neuronal differentiation. *Efna5* encodes for ephrinA5, and CBC treatment is able to reduce its expression in our NSC-34 cell model. Ephrins belong to a family of proteins that, during brain development, guide axonal projection using signals of attraction and repulsion. *Efna5* leads to the collapse of the axonal growth cone. In SH-SY5Y cells, *Efna5* contributed to the dopaminergic differentiation by reducing its mRNA expression [45], probably suppressing its axonal-repressor activity. In our experiment, after CBC exposure, the level of *Efna5* is reduced as well, suggesting the presence of a starting point towards the differentiation process. *Plp1* encodes for proteolipid protein 1, and it is upregulated in NSC-34 after CBC exposure. It is a component of myelin, and it is normally expressed in glutamatergic and cholinergic neurons of the brainstem, while its expression is not detectable in GABAergic neurons [46]. However, this information should not be interpreted as a suggestion that NSC-34 is starting to make myelin. Experiments on knockout animals for the *Plp1* gene revealed that this gene is not strictly necessary to develop a correct myelination, but its absence leads to axonal degeneration and axonal retrograde transport impairment [47]. *Plp1* is indeed necessary for the integrity of axons, so its overexpression after CBC treatment could be a good signaling for correct axonal growth.

*Neurod1* encodes for neuronal differentiation 1, and CBC treatment upregulates its expression in NSC-34 cells. *Neurod1* is a Basic helix-loop-helix transcription factor and, as another member of this family, contributes to the differentiation of various cell types. In the case of *Neurod1*, its main role is exerted during embryonic neurogenesis, and indeed it is also used to reprogram somatic cells into neuronal type. *Neurod1* is the base from which many other genes are expressed or repressed during neuronal differentiation, indicating *Neurod1* is one of the main players during this process [48]. The upregulation of *Neurod1* in our experimental set strongly indicates that CBC is able to trigger neuronal differentiation signaling in NSC-34. 

One receptor of acetylcholine, cholinergic receptor nicotinic alpha 1 subunit (AChRα1), is not present in experimental groups at 24 h, but its protein amount increases after 48 h, more in the nucleus than in cytoplasm, as shown in Figure 6E,F. The acetylcholine receptor is known to play a role during the neuronal differentiation of embryonic cells [49]; indeed, the modulation of AChR was demonstrated to be important in the differentiation of cells belonging to rodent neocortex [50]. However, these data need to be associated with *Ache* transcriptomic data. *Ache* encodes for acetylcholinesterase (AChE), and it is strongly upregulated after CBC treatment. AChE is the enzyme responsible for the degradation of acetylcholine, a neurotransmitter implied in cholinergic transmission. One important role of AChE is at the neuromuscular junction level, where it stops the cholinergic transmission from motor neurons to muscles [51]. Moreover, AChE is reported to be transcribed during neuronal differentiation [52], and indeed AChE interaction with lamin-1 promotes neurites outgrowth [53]. The increased expression of *Ache* in our experimental set is a sign of ongoing differentiation, and the fact that is expressed in motor neurons is in favor of our hypothesis. It is important to mention that the *Ache* gene is even more overexpressed after 48 h in NSC-34 treated with CBC. These pieces of evidence indicate that NSC-34 is preparing enzymes and neurotransmitters typical of cholinergic neurons, probably because of the ongoing differentiation. 

The evidence that CBC induces neuronal differentiation is also supported, apart from the mentioned genes expression, also by Western blot analysis of GFAP protein amount as follows: as shown in Figure 6A–D, after between 24 and 48 h, there is no expression of GFAP. GFAP is a protein highly specific for astroglial lineage cells [54]. The lack of GFAP protein suggests that, even after 48 h of CBC exposure, NSC-34 are not proceeding through astroglial differentiation. This is also supported by Figure 3 since the cells did not show any typical astrocyte morphology.

**Figure 7:**
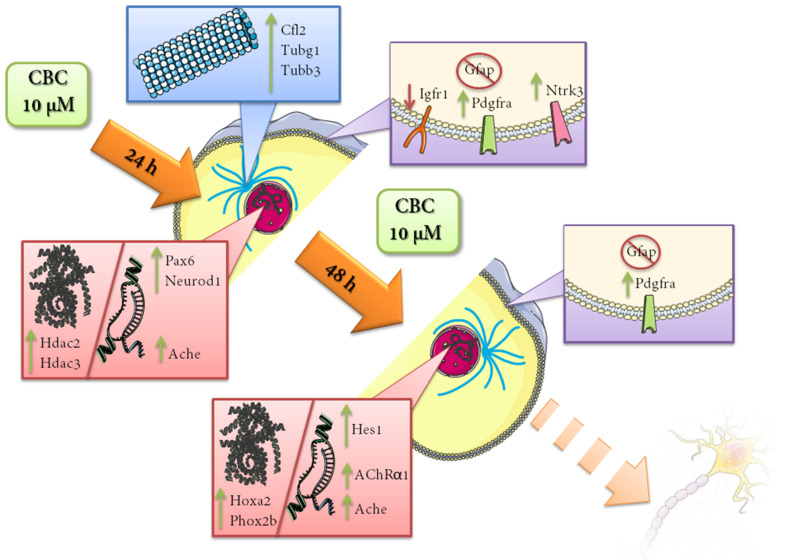
Main neuronal differentiation markers upregulated in NSC-34 after between 24 and 48 h of 10 µM of CBC treatment. CBC proved to be able to influence neuronal differentiation at different levels, increasing transcription factors aimed to develop mature neuron properties and also trigger signals for cytoskeleton remodeling. The influence on the expression of several receptors involved in neuronal differentiation supports the hypothesis. Green arrows indicate that the corresponding genes are upregulated, while red arrows indicate the genes that are downregulated.

The main evidence of our findings is resumed in Figure 7.

## 5. Conclusions

CBC proved to induce the expression of several genes correlated with the neuronal differentiation process in NSC-34, such as *Hoxa2*, *Pax6* and *Pum2*. Moreover, the increased expression of *Neurod1*, *Ache* and AChRα1 protein levels and *Tubb3* suggests the cell’s progression to a more mature stage of development toward cholinergic neurons. The use of CBC, as indicated by our results, could represent an important addition to the regeneration of the nervous system, but further experiments need to clarify and optimize how CBC could be efficiently used for this purpose.

## Figures and Tables

**Table 1:** Markers of neurodifferentiation differentially expressed against CBC.

Gene	CTRL vs. CBC Fold Change	*p*-Value	q-Value	CTRL vs. CBC-48 h Fold Change	*p*-Value	q-Value
*Ache*	1.83	1.03 × 10^−4^	5.16 × 10^−4^	2.03	4.91 × 10^−6^	2.46 × 10^−05^
*Cfl2*	0.24	1.52 × 10^−7^	1.44 × 10^−6^	−0.24	4.99 × 10^−8^	3.30 × 10^−07^
*Efna5*	−0.15	1.75 × 10^−2^	4.05 × 10^−2^	−0.16	3.59 × 10^−3^	9.33 × 10^−03^
*Hdac2*	0.33	1.05 × 10^−47^	1.82 × 10^−45^	0.11	7.56 × 10^−8^	4.88 × 10^−07^
*Hdac3*	0.14	2.21 × 10^−3^	7.08 × 10^−3^	0.29	2.32 × 10^−13^	2.54 × 10^−12^
*Hes1*	1.67	1.54 × 10^−4^	7.33 × 10^−4^	2.65	4.21 × 10^−11^	3.90 × 10^−10^
*Hoxa2*	2.23	2.18 × 10^−35^	2.38 × 10^−33^	1.16	1.61 × 10^−10^	1.41 × 10^−09^
*Igf1r*	−0.41	1.95 × 10^−15^	5.58 × 10^−14^	0.35	1.06 × 10^−17^	1.51 × 10^−16^
*Mest*	0.10	1.27 × 10^−2^	3.11 × 10^−2^	0.43	2.79 × 10^−33^	7.69 × 10^−32^
*Neurod1*	0.80	6.75 × 10^−6^	4.57 × 10^−5^	-	>0.05	>0.05
*Nrxn2*	0.13	7.25 × 10^−4^	2.77 × 10^−3^	0.37	5.24 × 10^−26^	1.08 × 10^−24^
*Ntrk3*	0.46	2.54 × 10^−5^	1.49 × 10^−4^	−0.92	1.03 × 10^−14^	1.21 × 10^−13^
*Pax6*	0.90	1.04 × 10^−38^	1.30 × 10^−36^	-	>0.05	>0.05
*Pdgfra*	2.01	1.47 × 10^−5^	9.09 × 10^−5^	2.7	4.46 × 10^−10^	3.74 × 10^−09^
*Phox2b*	0.35	4.68 × 10^−15^	1.29 × 10^−13^	0.26	3.29 × 10^−10^	2.80 × 10^−09^
*Plp1*	0.32	1.87 × 10^−2^	4.28 × 10^−2^	−0.32	1.57 × 10^−2^	3.29 × 10^−02^
*Pum2*	0.33	2.01 × 10^−61^	5.46 × 10^−59^	−0.2	1.57 × 10^−25^	3.16 × 10^−24^
*Smarce1*	0.29	1.19 × 10^−15^	3.51 × 10^−14^	−0.63	2.25 × 10^−67^	1.28 × 10^−65^
*Tubb3*	0.21	8.27 × 10^−109^	3.83 × 10^−106^	0.17	1.46 × 10^−88^	1.11 × 10^−86^
*Tubg1*	0.13	1.68 × 10^−7^	1.57 × 10^−6^	−0.16	1.61 × 10^−12^	1.64 × 10^−11^

Markers identified in the comparison of CBC against CTRL at 24 h or 48 h. CTRL and CBC columns show the level of expression of each marker in the specific condition after DESeq2 normalization. Fold change was calculated as log_2_ (CBC/CTRL). All the values are rounded to the second decimal digit.

## Data Availability

The data presented in this study are openly available in the NCBI Sequence Read Archive at BioProject accession number PRJNA907185.
